# Klippel-Trenaunay Syndrome with Extensive Lymphangiomas

**DOI:** 10.1155/2015/581394

**Published:** 2015-10-26

**Authors:** Sirin Mneimneh, Ali Tabaja, Mariam Rajab

**Affiliations:** Pediatric Department, Makassed General Hospital, Lebanon

## Abstract

Klippel-Trenaunay syndrome (KTS) is a rare disorder characterized by the triad of vascular malformations, venous varicosities, and bone and soft-tissue hypertrophy. We present a case of Klippel-Trenaunay syndrome with limb hypertrophy, port-wine stains, lymphangiomas, and venous varicosities in the limbs.

## 1. Introduction

Klippel-Trenaunay syndrome (KTS) was first described in 1900 by two French physicians, Klippel and Trenaunay [1]. The term describes a rare congenital syndrome of venous, lymphatic, and capillary malformations and soft tissue and bone hypertrophy [2].

63% of patients with KLS have the manifestation of the complete triad (port-wine stain, varicose veins, and hypertrophy of soft tissues/bones) [2]. Patients can be diagnosed with KTS with only one or more of the above-mentioned features since patient might not have all the features [3, 4].

Lymphangiomas are benign tumors of the lymphatic system. About 50% are present at birth and up to 90% become evident by the age of 2 years. Nearly 75% of the lesions are located on the head and neck (61%) or axilla (13%). The other 25% are distributed over the trunk (11%), extremities (11%), mediastinum (1%), and abdomen and genitalia (3%) [5].

We report a case of Klippel-Trenaunay with extensive lymphangioma throughout most of the body.

## 2. Case Report

**Figure 1:**
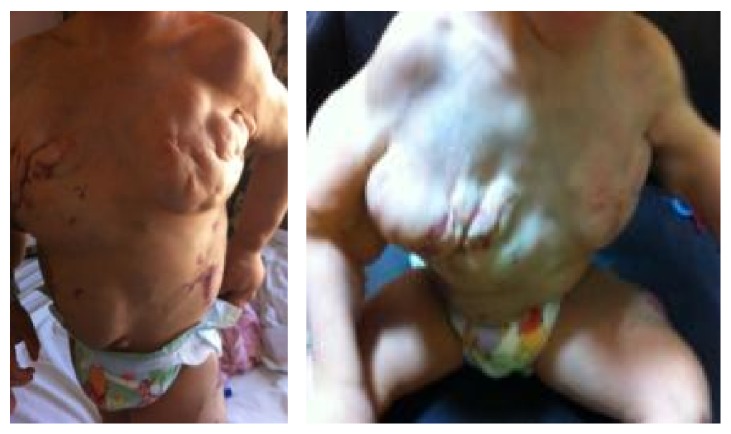
Focal overgrowth over the upper part of the chest.

**Figure 2:**
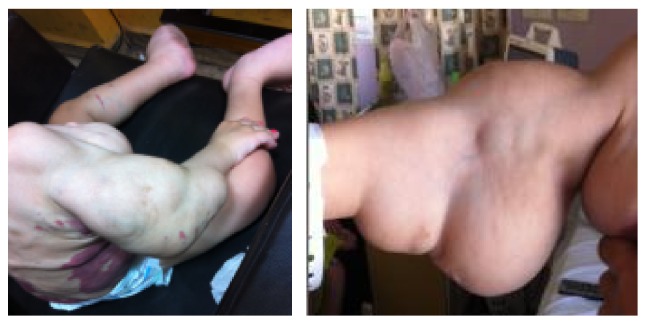
Multiple focal overgrowth of the right arm.

**Figure 3:**
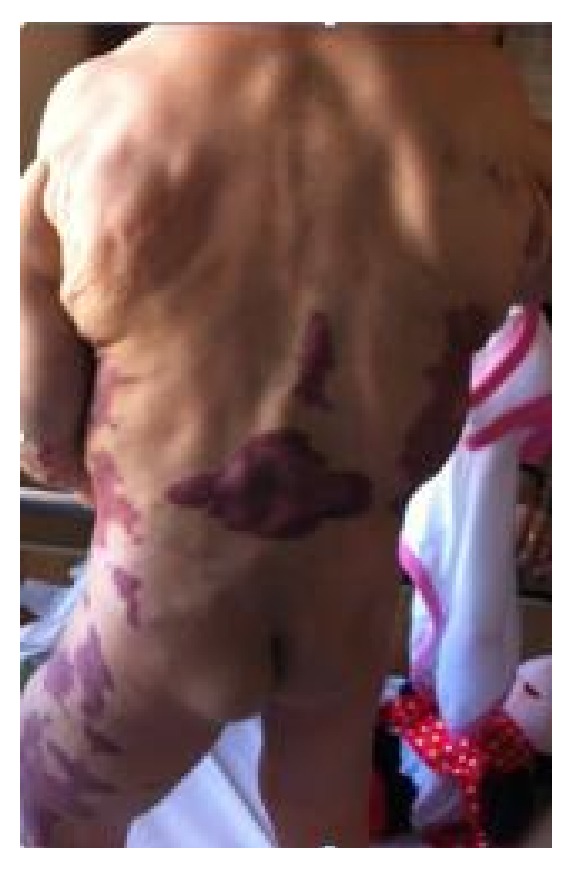
Port-wine stain over the back and thigh.

**Figure 4:**
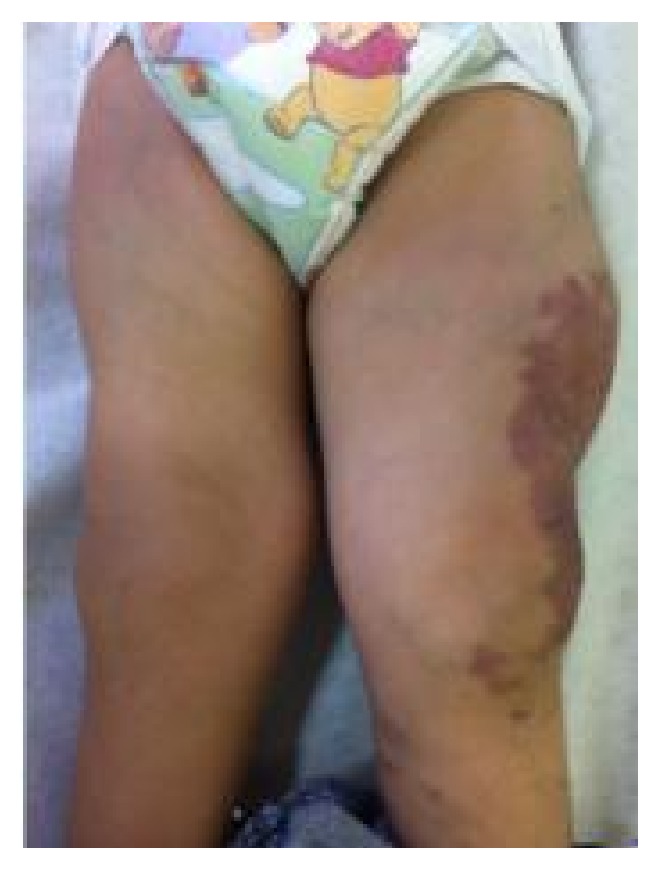
Hypertrophy of the left thigh.

**Figure 5:**
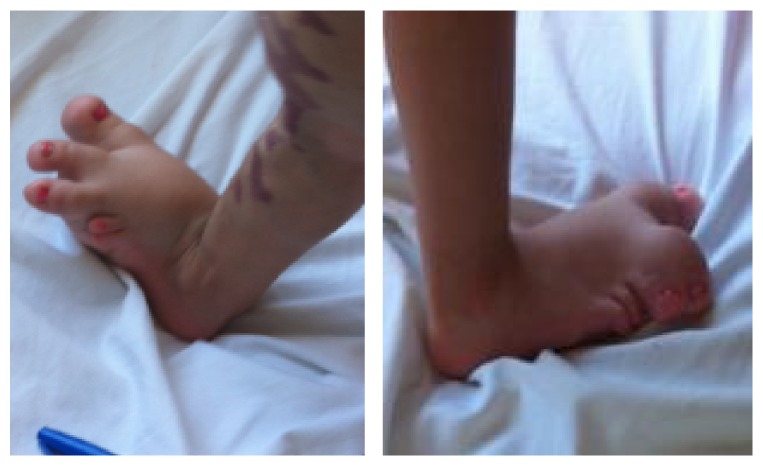
Syndactyly of the toes in both feet.

A 20-month-old girl presented since birth with multiple soft truncal masses that involved the anterior aspect of the upper chest, abdomen (Figure 1), and right arm (Figure 2). The body was partly covered by a large port-wine stain capillary malformation (Figure 3). Extremities showed hypertrophy of the left thigh (Figure 4) and overgrowth of both feet, with syndactyly of the second and third toes of the right foot (Figure 5). The patient had dilated tortuous veins over the chest and the left leg (venous varicosities).

The patient had no dysmorphic features or facial malformations; on examination she had no murmur, no organomegaly, good muscle power, and no deficits with positive deep tendon reflexes along with normal developmental milestones and normal growth parameters. She was the product of a nonconsanguineous marriage to a gravida three mother with two previous healthy children, born by Cesarean section (due to fetal malformations).

**Figure 6:**
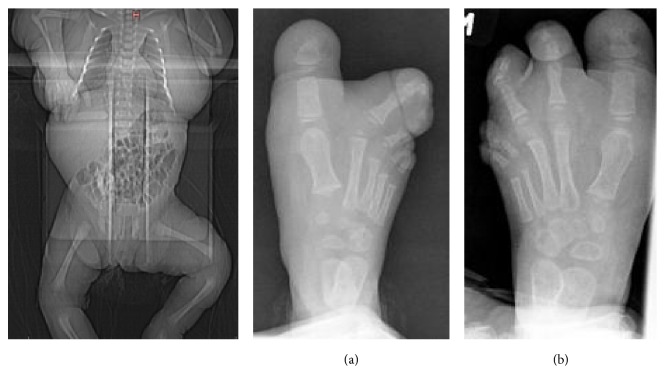
X-ray. (a) Right foot; (b) left foot.

Skeletal survey showed fused right second and third toes and hypertrophied soft tissue of these toes and of the right big toe. The patient also had fused left fourth and fifth toes and hypertrophied soft tissue of the left big toe (Figure 6).

**Figure 7:**
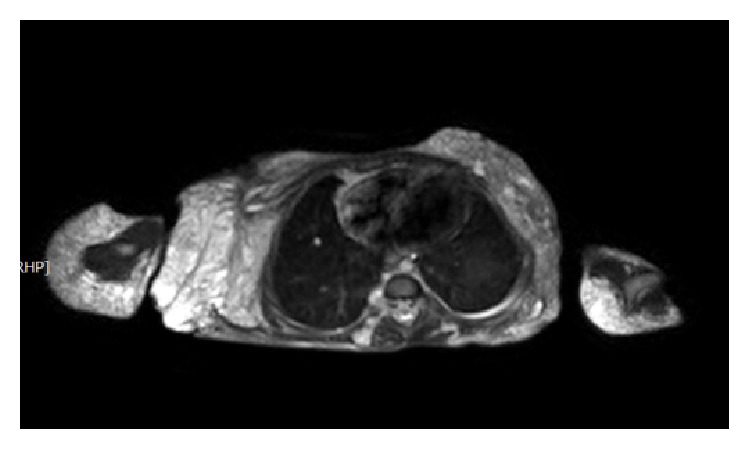
MRI of the chest (phase T2 sequence, with fat suppression), showed the lymphangioma in the upper part of the chest wall, in the right arm.

**Figure 8:**
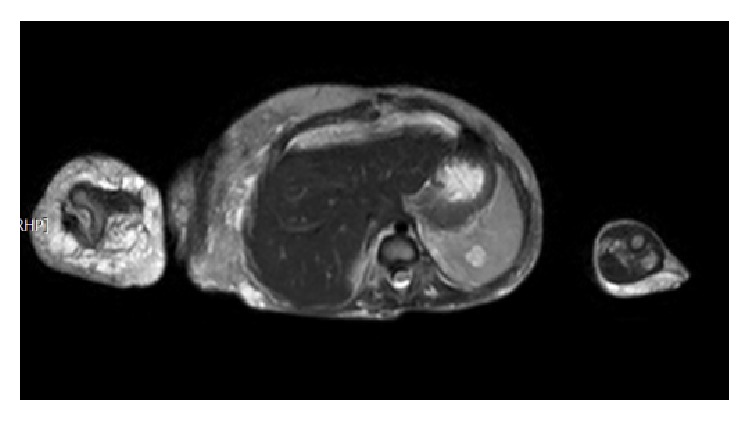
MRI of the abdomen showed the hemangioma in the spleen.

MRI examination of the chest, abdomen, and pelvis showed extensive lymphangioma of the following (the lymphangioma appeared on T2 as high signal masses mostly cystic with multiple septation): right upper limb, upper part of the chest, upper portion of the left lower limb, left side of the pelvis, and the retroperitoneum (Figure 7). The liver is of normal size and signal showing no focal lesions. The pancreas has a normal acinar pattern with 12.3 mm hemangioma, and the Wirsung duct is of normal caliber. The spleen is of normal size and signal showing small hemangioma measuring 12 mm (Figure 8). The kidneys appear normal with no hydronephrosis.

The parents preferred to go abroad in order to do the surgical excision.

## 3. Discussion

Longitudinal growth results from complex multifactorial processes that take place in the broader context of different genetic traits and environmental influences. Overgrowth syndromes include a heterogeneous group of disorders that lead to excessive tissue proliferation, which is characterized by a phenotype of excessive visceral and somatic growth [6].

Klippel-Trenaunay syndrome is rare with uncertain origin with an incidence of approximately 1 : 100,000 live births [7]. It has no predilection for gender or race, and most of the cases are sporadic and appear at birth [8]. The French physicians Maurice Klippel and Paul Trenaunay first described this syndrome in 1900 when they associated vascular malformations with hypertrophy in the affected limb. Subsequently, Parkes Weber described arteriovenous fistulas in these patients [8].

The etiology of the disease is still under investigation. Some hypothesized that embryonic mesodermal changes resulting in increased angiogenesis lead to increased vascular flow causing tissue hypertrophy and vascular changes [9]. Others agree that the majority of cases of KTS are due to sporadic polygenic mutations [10]. The association between the angiogenic factor gene* AGGF1* and KTS appears to be significant [11].

KTS is characterized by the presence of capillary malformations associated with venous malformations or varicose veins and with bone or tissue hypertrophy [12].

Skin malformations in KTS are mostly capillary hemangiomas. Skin capillary malformations are diffuse or mostly located on the hypertrophic extremity side. Lower extremities are affected in about 95% of cases [3]. Changes can be limited to the skin only or can affect subcutaneous tissue, muscles, and bones. 56% of patients have visceral vascular malformations including hemangiomas and/or lymphangiomas [13]. The spleen is rarely affected in patients with KTS and it can appear as splenomegaly, hemangioma, and/or lymphangioma of the spleen [14].

In a study at the Mayo Clinic, port-wine stains were seen in 98% of patients, varicosities or venous malformations in 72%, and limb hypertrophy in 67%. Atypical veins, including lateral veins and persistent sciatic veins, were present in 72% [15].

In a study of 144 patients, 95% had a cutaneous vascular malformation, 93% had soft tissue and bone hypertrophy, 76% had varicosities, and 71% had involvement limited to one extremity [16].

Sometimes lymphangiomas are associated with lymphangiomatosis located in the mediastinum, retroperitoneum, axilla, or neck. Lymphangiomas are rarely unilocular or more often, as in this case, made of numerous septated cystic areas [17].

Our patient presented directly after birth with focal soft masses over the chest, hypertrophy of the soft tissues of lower extremities, associated with vascular malformation (port-wine stain) over the skin, and the hemangiomas in the internal organs. The extensive lymphangiomas make our case peculiar and distinguish it from other reported cases.

Complications are most often related to the underlying vascular pathological condition. Vascular malformations involving the gastrointestinal and genitourinary tract are a significant source of morbidity and even mortality. Involvement of the gastrointestinal tract occurs in 20% of the patients, can present at any age, and may go unrecognized in patients without overt symptoms.

Pulmonary embolism, cerebral aneurysm, and pulmonary vein varicosities are incidental findings and give rise to life-threatening complications [18].

Regular clinical and radiographic monitoring of the affected limbs, compression stockings for chronic venous insufficiency, intermittent pneumatic compression devices for reducing limb size, flash lamp-pumped pulsed dye laser for port-wine stains, and surgical correction of varicose veins are needed as required [19].

Surgical debulking often fails or worsens symptoms as venous and lymphatic channels are destroyed, leading to further swelling and poor wound healing. Overall, treatment is often not definitive and 50% of patients reexperience symptoms after surgery despite reported clinical and symptom severity improvement in many patients.

The main differential diagnosis includes the following.

Proteus syndrome is characterized by massive overgrowth and asymmetry. The types of skin lesions include linear verrucous epidermal nevi, intradermal nevi, hemangiomas, lipomas, and varicosities [20]. Macrodactyly and syndactyly can occur; although final height is normal, increased stature in childhood is common in addition to macrocephaly. Soft tissue hypertrophy, which may appear as gyriform changes, is mainly over the planter surface. Moderate mental deficiency occurs in approximately 50% of cases. Bone overgrowth, which is dysplastic, progressive, and irregular, is typical of Proteus syndrome and not observed in KTS; thus, its detection is an important tool in differentiating between the diseases. Our patient does not represent the typical skin lesions; her mental milestones are normal as well as her height, weight, and head circumference measurements.

Maffucci syndrome is characterized by multiple enchondromas, hemangiomas and, less often, lymphangiomas. Enchondromas are cartilaginous benign tumors that may develop most frequently in phalanges and long bones, but may also affect the tibia, fibula, humerus, ribs or cranium. Soft tissue tumors usually develop with the bone lesions [21].

Neurofibromatosis type I generally affects the skin, nervous system, bones, and endocrine glands by causing benign tumors. The diagnostic criteria for this disease were developed in 1987 and redefined in 1997 [22] and they are based on the presence of two or more of the following findings: a first-degree relative who has neurofibromatosis type I, “café-au-lait” spots, neurofibromas, freckles in the axillary or inguinal regions, optic gliomas, iris hamartomas, and distinctive bone lesions.

Sturge-Weber syndrome is a mesodermal phakomatosis characterized by a port-wine vascular nevus on the upper part of the face, leptomeningeal angiomatosis that involves one or both hemispheres, early onset seizure, choroidal vascular lesions associated with glaucoma, neurologic deterioration, and eventual neurodevelopmental delay [23].

Schnyder et al. [24] suggested that Klippel-Trenaunay, Parkes-Weber, and Sturge-Weber syndrome are variants with a common feature of local gigantism and hemangioma. Involvement of the head and neck with manifestations of meningeal angiomatosis causing epileptic fits and choroidal hemangioma or glaucoma result in a presentation of Sturge-Weber syndrome. Local gigantism and hemangioma of the limbs with osteohypertrophy present as Klippel-Trenaunay or Parkes-Weber syndrome, depending on whether there are peripheral varices which cause KTS or arteriovenous anastomoses which would result in a picture of Parkes-Weber syndrome.

In summary, Klippel-Trenaunay syndrome is a rare disease of the vascular and lymphatic system or capillary malformation at birth associated with hypertrophy of the soft tissues/bones. Most cases are difficult to treat due to high rates of recurrence after surgical excision, but individualized intervention can help manage pain and help prevent serious complications.
